# Supplementary material for: Identification of Freezing-Responsive microRNAs and Their Targets in Chinese Jujube by Small RNA and Degradome Sequencing
Source: Int J Mol Sci. 2026 Sep 4;27(17):7912. doi: 10.3390/ijms27177912 (PMC13566890; doi:10.3390/ijms27177912)
Supplement: Supplementary file 1 [file ijms-27-07912-s001.zip › Supplementary Material S2/novel_miR_223.pdf]

Provisional ID	:	novel_miR_223
Score total	:	4.2
Score for star read(s)	:	-1.3
Score for read counts	:	0
Score for mfe	:	3.9
Score for randfold	:	1.6
Score for cons. seed	:	
Total read count	:	212
Mature read count	:	189
Loop read count	:	17
Star read count	:	6

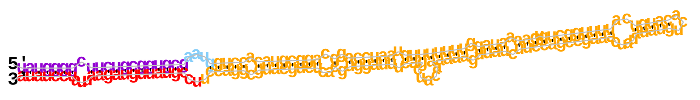

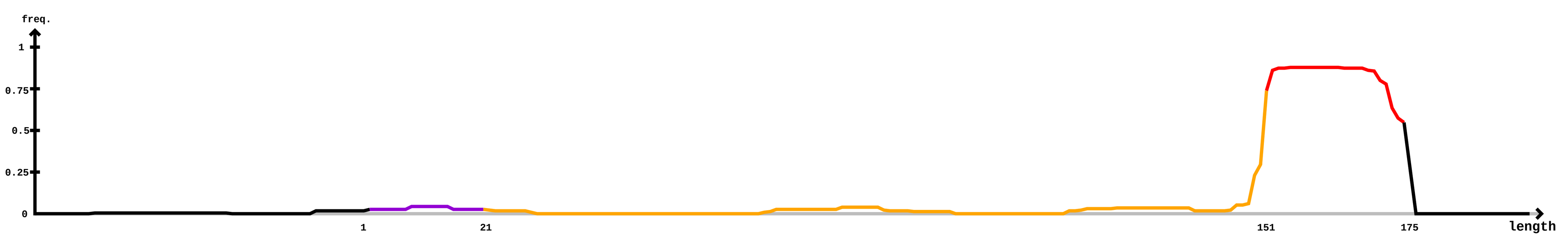

Star Mature

```
5'- ugauaauaugcuauaugugauacuaacucuuuuauauauauauauauauauauauauggggcuucugcggugcgaauggucccacaugcggacggaccuaauguuuuuuggauaaaagugucgguuuuacuguacacuguauauaucaaagccgaccuucuauugaaaaaacaucgacuuuaggugcacucugcaugcggaccuuccguauuguagaauuauccuauauauauauauauauauauauaugca -3' obs
    ugauaauaugcuauaugugauacuaacucuuuuauauauauauauauauauauauauggggcuucugcggugcgaauggucccacaugcggacggaccuaauguuuuuuggauaaaagugucgguuuuacuguacacuguauauaucaaagccgaccuucuauugaaaaaacaucgacuuuaggugcacucugcaugcggaccuuccguauuguagaauuauccuauauauauauauauauauauauaugca     exp
```

read	reads	mm	sample
ugcuauaugugauacuaacucuu	1	0	LT-2
auauauauauaugggggcuucugc	1	0	JCK-2
auauauauauaugggggcuucugc	3	0	JCK-3
uauggggcuucugcggugcg	1	0	JCK-3
uauggggcuucugcggugcg	1	0	JCK-2
cuucugcggugcgaaugguc	1	0	JCK-2
cuucugcggugcgaaugguc	1	0	JCK-3
cuucugcggugcgaauggucc	1	0	JCK-3
cuucugcggugcgaauggucc	1	0	JCK-1
ucggCuuuacuguacacugu	1	1	LT-3
ucgAuuuuacuguacacugua	1	1	JCK-3
cgguuuuacuguacacuguauaua	1	0	JCK-2
gAuuuuacuguacacugu	3	1	LT-2
acacuguaCauaucaaagc	3	1	LT-2
caucgacuuuaggugcacucu	1	0	JCK-3
caucgacuuuaggugcacucu	1	0	JCK-2
caucgaAuuuaggugcacucu	1	1	JCK-3
caucgacuuuaggugcacucu	1	0	JCK-1
ucgacuuuaggugcacucugcaugcU	1	1	LT-3
cgacuuuaggugcacucugcaugc	1	0	JCK-1
cgacuuuaggugcacucugcaugc	1	0	JCK-3
uuaggugcacucugcaugcggacc	1	0	JCK-3
ggaccuuccguauuguaga	1	0	LT-3
ggaccuuccguauuguagaauua	1	0	JCK-3
ggaccuuccguauuguagaauua	1	0	JCK-1
gaccuuccguauuguagaauuauc	2	0	JCK-2
gaccuuccguauuguagaauuauc	1	0	JCK-3
gaccuuccguauuguagaauuauc	1	0	JCK-1
gaccuuccguauuguagaauuaucA	1	1	JCK-3
gaccuuccguauuguagaauuaucA	1	1	JCK-2
gaccuuccguauuguagaauuaucA	1	1	LT-3
gaccuuccguauuguagaauuaucA	1	1	JCK-1
ccuuccguauuguagaauuau	1	0	LT-2

```
                                                      Star                                                                                                                                                                        Mature
ugauaauaugcuauaugugauacuaacucuuuuauauauauauauauauauauauauggggcuucugcggugcgaaugguccacaugcggacggaccuaauguuuuuuuggauaaaagugucgguuuuacuguacacuguauauaucaaagccgaccuucuauugaaaaaacaucgacuuuaggugcacucugcaugcggaccuuccguauuguagaauuauccuauauauauauauauauauauaugca
.........................................................................................................................................................................................................ccuuccguauuguagaauuaucA..........................	1	1	JCK-3
..........................................................................................................................................................................................................cuuccguauuguagaauua.............................	1	0	JCK-3
..........................................................................................................................................................................................................cuuccguauuguagaauuauccA.........................	9	1	JCK-2
..........................................................................................................................................................................................................cuuccguauuguagaauuauccA.........................	4	1	JCK-3
..........................................................................................................................................................................................................cuuccguauuguagaauuauccA.........................	5	1	JCK-1
..........................................................................................................................................................................................................cuuccguauuguagaauuauccA.........................	1	1	LT-3
..........................................................................................................................................................................................................cuuccguauuguagaauuauccA.........................	12	1	LCK-3
..........................................................................................................................................................................................................cuuccguauuguagaauuauccu.........................	1	0	LT-3
..........................................................................................................................................................................................................cuuccguauuguagaauuauccu.........................	1	0	JCK-1
..........................................................................................................................................................................................................cuuccguauuguagaauuauccua........................	2	0	JCK-1
..........................................................................................................................................................................................................cuuccguauuguagaauuauccua........................	2	0	JCK-2
..........................................................................................................................................................................................................cuuccguauuguagaauuauccua........................	1	0	JCK-3
...........................................................................................................................................................................................................uuccguauuguagaauuauc...........................	1	0	JT-3
...........................................................................................................................................................................................................uuccguauuguagaauuauc...........................	2	0	JCK-3
...........................................................................................................................................................................................................uuccguauuguagaauuauc...........................	1	0	JT-2
...........................................................................................................................................................................................................uuccguauuguagaauuauc...........................	1	0	JCK-2
...........................................................................................................................................................................................................uuccguauuguagaauuauc...........................	3	0	LT-3
...........................................................................................................................................................................................................uuccguauuguagaauuauccua........................	1	0	JCK-1
...........................................................................................................................................................................................................uuccguauuguagaauuauccua........................	2	0	JCK-3
...........................................................................................................................................................................................................uuccguauuguagaauuauccua........................	3	0	LT-2
...........................................................................................................................................................................................................uuccguauuguagaauuauccuaC.......................	1	1	JCK-1
...........................................................................................................................................................................................................uuccguauuguagaauuauccuaC.......................	1	1	JCK-3
............................................................................................................................................................................................................uccguauuguagaauuauccuau.......................	1	0	JCK-3
............................................................................................................................................................................................................uccguauuguagaauuauccuaua......................	3	0	JT-1
............................................................................................................................................................................................................uccguauuguagaauuauccuaua......................	1	0	LT-2
............................................................................................................................................................................................................uccguauuguagaauuauccuaua......................	38	0	JCK-3
............................................................................................................................................................................................................uccguauuguagaauuauccuaua......................	32	0	JCK-2
............................................................................................................................................................................................................uccguauuguagaauuaGccuaua......................	1	1	JCK-1
............................................................................................................................................................................................................uccguauuguagaauuauccuauG......................	1	1	JCK-2
............................................................................................................................................................................................................uccguauuguagaauuauccuaua......................	1	0	LT-1
............................................................................................................................................................................................................uccguauuguagaauuauccuaua......................	6	0	LCK-3
............................................................................................................................................................................................................uccguauuguagaauuauccuaua......................	18	0	JCK-1
.............................................................................................................................................................................................................ccguauuguagaauuauc...........................	1	0	LT-3
.............................................................................................................................................................................................................ccguauuguagaauuauccuau.......................	1	0	JCK-3
.............................................................................................................................................................................................................ccguauuguagaauuauccuau.......................	2	0	JCK-2
.............................................................................................................................................................................................................ccguauuguagaauuauccuauG......................	1	1	JCK-1
.............................................................................................................................................................................................................ccguauuguagaauuauccuauG......................	1	1	JCK-2
.............................................................................................................................................................................................................ccguauuguagaauuauccuaua......................	11	0	JCK-2
.............................................................................................................................................................................................................ccguauuguagaauuauccuaua......................	5	0	JCK-1
.............................................................................................................................................................................................................ccguauuguagaauuauccuaua......................	6	0	JCK-3
..............................................................................................................................................................................................................cguauuguagaauuauccuG........................	3	1	LT-2
................................................................................................................................................................................................................uauuguagaauuauccuaua......................	1	0	LT-2
```
